# The Prospects of *Swietenia macrophylla* King in Skin Care

**DOI:** 10.3390/antiox11050913

**Published:** 2022-05-06

**Authors:** Camille Keisha Mahendra, Khang Wen Goh, Long Chiau Ming, Gokhan Zengin, Liang Ee Low, Hooi-Leng Ser, Bey Hing Goh

**Affiliations:** 1 Biofunctional Molecule Exploratory Research Group, School of Pharmacy, Monash University Malaysia, Bandar Sunway 47500, Malaysia; camille.mahendra@monash.edu; 2 Faculty of Data Science and Information Technology, INTI International University, Nilai 71800, Malaysia; khangwen.goh@newinti.edu.my; 3 PAP Rashidah Sa’adatul Bolkiah Institute of Health Sciences, Universiti Brunei Darussalam, Gadong BE1410, Brunei; 4 Biochemistry and Physiology Research Laboratory, Department of Biology, Science Faculty, Selcuk University, Konya 42130, Turkey; gokhanzengin@selcuk.edu.tr; 5 Chemical Engineering Discipline, School of Engineering, Monash University Malaysia, Bandar Sunway 47500, Malaysia; low.liangee@monash.edu; 6 Advanced Engineering Platform, Monash University Malaysia, Bandar Sunway 47500, Malaysia; 7 Department of Biological Sciences, School of Medical and Life Sciences, Sunway University, Bandar Sunway 47500, Malaysia; hooilengs@sunway.edu.my; 8 Novel Bacteria and Drug Discovery Research Group, Microbiome and Bioresource Research Strength Jeffrey Cheah School of Medicine and Health Sciences, Monash University Malaysia, Bandar Sunway 47500, Malaysia; 9 College of Pharmaceutical Sciences, Zhejiang University, 866 Yuhangtang Road, Hangzhou 310058, China; 10 Health and Well-Being Cluster, Global Asia in the 21st Century (GA21) Platform, Monash University Malaysia, Bandar Sunway 47500, Malaysia

**Keywords:** cosmeceutical, photoaging, anti-pigmentation, *Swietenia macrophylla*, natural product, skin whitening, skin repair and regeneration, wound healing, acne vulgaris, anti-microbial

## Abstract

The importance of cosmetics in our lives is immeasurable. Covering items from daily personal hygienic products to skincare, it has become essential to consumers that the items that they use are safe and effective. Since natural products are from natural sources, and therefore considered “natural” and “green” in the public’s eyes, the rise in demand for such products is not surprising. Even so, factoring in the need to remain on trend and innovative, cosmetic companies are on a constant search for new ingredients and inventive new formulations. Based on numerous literature, the seed of *Swietenia macrophylla* has been shown to possess several potential “cosmetic-worthy” bioproperties, such as skin whitening, photoprotective, antioxidant, antimicrobial, etc. These properties are vital in the cosmetic business, as they ultimately contribute to the “ageless” beauty that many consumers yearn for. Therefore, with further refinement and research, these active phytocompounds may be a great contribution to the cosmetic field in the near future.

## 1. Introduction

Cosmetics, since time immemorable, have always been a large part of our lives. With global sales revenue projected to achieve $429.8 billion by 2022, the cosmetic industry is a lucrative, fast-paced, and innovative business that constantly seeks to fulfill consumer’s expectations and preferences [1,2]. Although many do not know it, the umbrella of cosmetics actually covers a large range of products categorized as personal hygiene, makeup, fragrances, skincare, and hair care [3]. Nevertheless, in the last few decades, the trend for “natural”, “green”, “sustainable”, and “safe” cosmetics is on the rise. With rising awareness and concerns regarding the ingredients within a product, there is an inclination for cosmetics that are developed from a natural source. This forces the cosmetic industry to be quick in its search for new, inventive, and potent natural ingredients that not only entice the consumers but also produce the desired effect as marketed [4]. One category that is highly affected by this recent trend is skincare. Natural product-based skincare products containing bioproperties, such as antioxidant, antibacterial, anti-inflammatory, whitening, and much more, are high in demand to combat skin aging and acne blemishes [5,6]. Based on these criteria, the properties of *Swietenia macrophylla* that were previously reported may prove to be an interesting, novel natural ingredient that can contribute to the development of skincare cosmetics. For an introduction, *S. macrophylla*, along with its congeners *Swietenia mahagoni* (L.) Jacq and *Swietenia humilis* Zucc., belong to the *Meliaceae* family. Easily found within the tropical region from North to South America, it is well known for its high-quality wood, used in the creation of furniture, artisanal crafts, decorative materials, and musical instruments [7,8]. Other than being a prized timber, its seed is also employed as a traditional medicine against various ailments and discomforts, such as hypertension, persistent pain, diabetes, malaria, etc. [9].

## 2. An Antimicrobial Remedy against Skin Disorders

Our skin is home to diverse microorganisms, such as bacteria, viruses, yeast, mites, fungi, etc. It is also through the mutual benefit and balance of the skin microbiota that our skin remains healthy. A healthy skin microbiota predominately consists of the following bacterial phyla: *Actinobacteria*, *Firmicutes*, *Proteobacteria*, and *Bacteroidetes* [10,11]. However, the imbalance in cutaneous microbiota, also known as dysbiosis, is frequently associated with the development of skin diseases, such as acne vulgaris [12]. By definition, acne vulgaris is a form of dermatoses that forms excess production of sebum, inflamed pilosebaceous units, and follicular hyperkeratinization, in the presence of selected phylotypes of *Cutibacterium acnes* (which was formerly known as *Propionibacterium acnes*) [12,13]. These bacteria thrive on sebum and are responsible for the release of proinflammatory cytokines that induce the occurrence of acne vulgaris [14]. In the meantime, another commensal bacterium, *Staphylococcus epidermidis*, fails to perform its role as a regulator of the skin’s homeostasis, hypothetically due to its decreased abundance that intensifies the disequilibrium in the skin microbiota [15]. Finally, accompanying the disruption of the skin barrier, opportunistic pathogens, such as *Staphylococcus aureus*, then aggravate the symptoms by promoting skin inflammation [15]. Thus, it is essential to retain the delicate balance of microbiota on our skin. This is where antibacterial cosmetic products come into play in the treatment of skin pathology. Besides treatment against the aforementioned bacteria during dysbiosis, it should also restore the equilibrium of the skin’s microbiota.

In a study conducted by Suliman and the team, it had been shown that the crude alkaloid extract of the seed can inhibit the growth of *S. aureus*, *Escherichia coli*, and *Pseudomonas aeruginosa*, with minimum bactericidal concentration (MBC) values of 12.5 mg/mL against *S. aureus* and 50 mg/mL against *E. coli* and *P. aeruginosa*. An almost similar value was obtained for the minimum inhibition concentration (MIC) assay, indicating that the extract is both inhibitory and bactericidal [16]. Interestingly, another study conducted on *P. aeruginosa* using *Caenorhabditis elegans* as a model showed that, although the seed extract improved its survival, it was unable to inhibit its growth and colonization in the worm’s gut. It was suggested that this observation could be attributed to additional effects of the extract, such as modifying the virulence factors of the associated bacteria or improving the host’s tolerance (as lysozyme-like protein 7 gene expression was increased) [17]. Although different outcomes were observed in both studies, it should be noted that different extraction solvents were used in the extraction process. Therefore, different types or amounts of phytocompounds could be extracted or excluded based on their polarity and solubility. Similarly, a study on the oil of the *S. macrophylla* seed, obtained through n-hexane and diethyl ether, also exhibited different levels of antagonistic effects towards the same bacterial species, with the addition of *Salmonella* Typhimurium [18]. In this case, there were smaller inhibition zones for both *S. aureus* and *P. aeruginosa* and no signs of inhibition against *E. coli* after treatment with the oil. Next, methanol extract of *S. macrophylla* seeds inhibited the growth of five different bacteria species and one yeast. These microorganisms were *Acinobacter anitratus*, *S. aureus*, *Bacillus cereus*, *Bacillus subtilis*, *Shigella boydii*, and *Candida utilis* [19]. Again, *E. coli* was not reported to be inhibited by this particular extract, which is the opposite to another study that also used methanol as the extracting solvent [19,20]. In an article written by Durai and team [20], both *S. aureus*, *E. coli*, and three other fungi (*Fusarium* sp., *Alternaria* sp., and *Helminthosporium* sp.) were inhibited by the methanol extract of *S. macrophylla*. The probable answer to this varying inhibitory activity on *E. coli* could be either the difference in strain or location of seed harvest. Regardless, even though methicillin-resistant *S. aureus* (MRSA) growth was not affected by *S. macrophylla* methanol extract, its virulence was suspected to decrease as the survival of *C. elegans* was increased after treating with 200 μg/mL of either methanol, ethyl acetate, or butanol extract [19,21].

**Figure 1:**
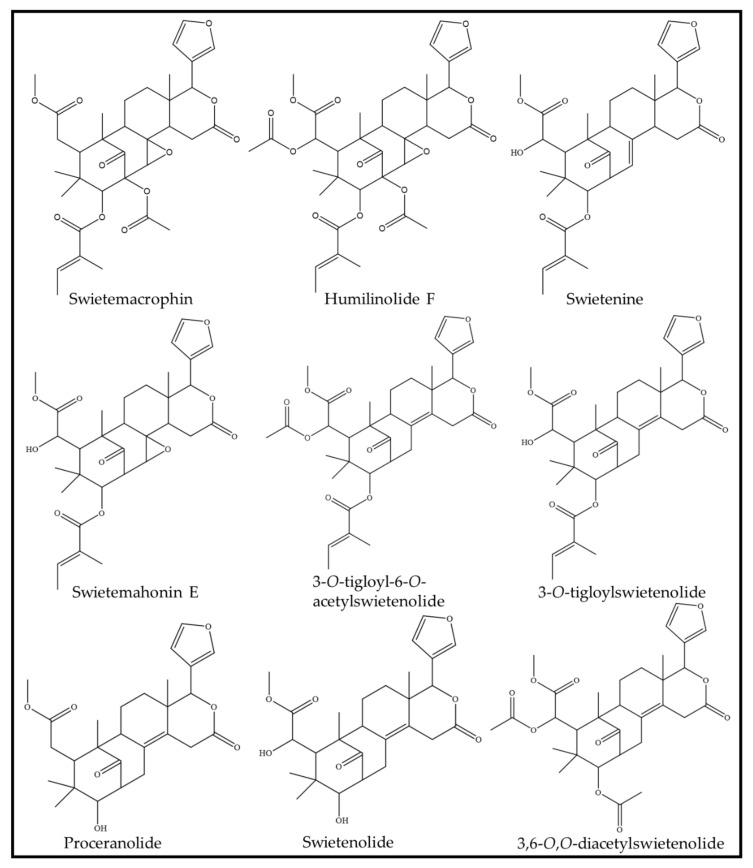
Limonoid phytocompounds extracted from *S. macrophylla* seed with cosmeceutical bioproperties.

After the purification of the extract, the antibacterial activity of *S. macrophylla* can be accredited to the presence of specific phytocompounds, as can be observed in Figure 1. One such phytocompound is swietenolide, a limonoid phytocompound of the triterpenoid class that can be found in the seed of *S. macrophylla.* In an early study conducted in 2009, the compound demonstrated antibacterial activity against eight different multiple-drug-resistant bacterial strains. These bacterial strains were group A β-haemolytic *S. aureus* (code 312), *S. aureus* (code 477), *Streptococcus pneumonia* (code 32215), *Haemophilus influenza* (code 32139), *E. coli* (code 169), *Klebsiella pneumonia* (code 32140), *Salmonella typhi* (code 1467), and *Salmonella paratyphi* (code 1272) [22]. Besides swietenolide, swietenine and 3-*O*-tigloylswietenolide were also reported to exhibit antibacterial activity against several Gram-positive and -negative bacteria. These bacteria included *B. subtilis*, *Bacillus megaterium*, *Sarcina lutea*, *S. aureus*, *E. coli*, *P. aeruginosa*, *S. typhi*, *S. boydii*, *Shigella dysenteriae*, *Shigella sonnei*, *Shigella shiga*, and *Klebsiella* sp. [23]. Looking at the similarities in the bacterial species tested across the different studies, it can be concluded that these three phytocompounds are indeed responsible for the antibacterial action of *S. macrophylla*. Interestingly, proceranolide, another limonoid that can be found in the seed, was discovered to have moderate inhibitory effects against two *Mycobacterium tuberculosis* strains (low virulent H37Rv ATCC27294 strain and high virulent M299 Beijing strain), with a MIC_50_ value of 37.6 ± 0.4 μg/mL and 44.9 ± 1.0 μg/mL for the H37Rv and M299 strains, respectively [24,25]. Although both bacteria strains are not of cosmeceutical importance, it could be beneficial for proceranolide to be included in future tests, in order to study its protective effects against pathogenic skin bacteria.

Overall, it is clear that the *S. macrophylla* seed does possess antimicrobial properties, as can be observed in Table 1. However, the strength and coverage of its antimicrobial activity are very much dependent on the presence of certain phytochemicals and their amount in the extract. That in itself pertains to many uncontrollable factors, such as extraction method, solvents used, location of the plant, and harvest seasons that affect the abundance of antimicrobial phytochemicals [26]. On the contrary, the inconsistency in the data reported could also be due to the dissimilar bacteria strains used. Among the articles reviewed, some studies did not reveal the strain number used, which challenges any form of direct comparison across articles. Therefore, for future improvements, it is encouraged that similar strains of bacteria, especially clinical strains, should be used in experiments to ease comparison across studies. It would also be useful to set a standard set of antimicrobial tests in the antimicrobial testing for cosmetic products. For example, the natural product incorporated in cosmetic creams and gels must be able to exhibit a certain degree of activity before these claims can be placed on the product labels. This is to ensure that the reported antimicrobial activity is up to par and that consumers would benefit from the desired effect (as claimed in the product description/labelling). Lastly, as of yet, *S. macrophylla* extracts have not been tested against some bacteria, such as *C. acnes* and *S. epidermidis*. To truly explore its use in cosmetics, it would be indispensable that the extracts and phytocompounds extracted are not only analyzed for their antimicrobial activity against them, but also if the extract and compounds can restore and promote the balance of the skin microbiota. Incidentally, it would be worthwhile to study the effect of *S. macrophylla* on bacteria-induced inflammation in skin diseases. Thus, this calls for antimicrobial studies beyond just the bacteria itself, such as in vivo analysis of the effect of *S. macrophylla* on the skin microbiome among the human population and clinical trials of *S. macrophylla* based skin care products.

## 3. Photoprotective, Skin Whitening, and Skin Repair Cosmeceutical Prospects of *S. macrophylla*

**Figure 2:**
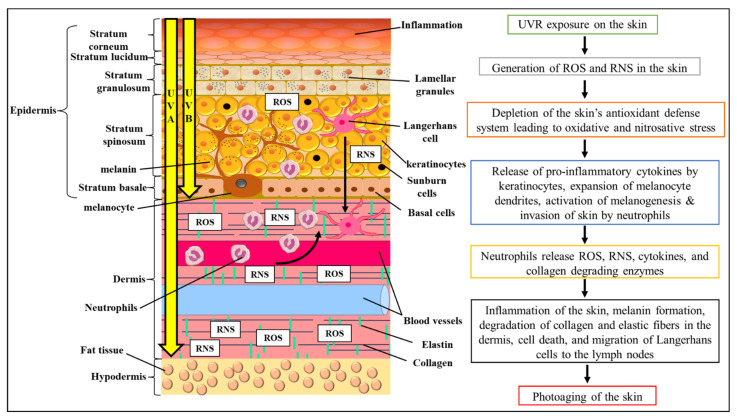
The impact of UVR exposure on the skin and how it mediates photoaging.

Aside from its antimicrobial properties, *S. macrophylla* seed extract has the potential for cosmetic use against skin aging. In spite of the many factors that can expedite the rate of skin aging, one main factor is the constant exposure of one’s skin to sunlight. Sunlight that can penetrate through the ozone layer contains ultraviolet A (UVA) and B (UVB). The differences between both types of ultraviolet rays (UVR) are their wavelength and penetration level through the skin. UVA, which has a longer wavelength of 320–400 nm, can penetrate deeply into the dermal layer of the skin. On the other hand, UVB, which has a shorter wavelength of 280–320 nm, can only penetrate up to the epidermal layer of our skin [27]. Regardless of their characteristics, overexposure to both UVR causes dysregulation in the skin’s circadian rhythm and activates the production of reactive oxygen species (ROS) and reactive nitrogen species (RNS) [28,29,30]. The overproduction of both ROS and RNS then quickly overwhelms the skin’s antioxidant defense system, causing nitrosative and oxidative stress in the skin [29]. In response, the skin begins producing pro-inflammatory cytokines, signaling the start of skin inflammation [31,32]. Following this, neutrophils quickly infiltrate the skin and secrete neutrophil elastase, which cleaves extracellular matrices and activates matrix metalloproteases [33,34,35]. Skin melanogenesis is also activated in response to UVR exposure by the activation of p53 expression, which goes on to initiate the transcription of the pro-opiomelanocortin (POMC) gene, eventually beginning the conversion of tyrosine into melanin [36]. Other events, such as the migration of Langerhans cells from the epidermis to the lymph nodes, were noted after UVR exposure. A side effect of this is that the skin’s hypersensitivity to allergens will be lowered during the event [37]. Direct DNA damage by UVB, through the formation of cyclobutene pyrimidine dimers (CPD) and pyrimidine-pyrimidone (6–4) photoproducts, also causes cell death and increases the risk of skin cancer [38,39,40]. A summary of the mentioned UVR-induced damage can be observed in Figure 2. According to this evidence, it becomes even more necessary to protect our skin against overexposure to UVR.

Photoprotection against UVR can be achieved through either immediate “blocking” of UVR penetration through the skin, or by reversing the activated pathways instigated through UVR exposure. The “blocking” of UVR penetration can be achieved through the application of either physical or chemical sunscreen. Physical sunscreens are sunscreens that contain titanium dioxide or zinc oxide nanoparticles that reflect and scatter UVR, whereas chemical sunscreens are made of organic molecules that absorb UVR energy and emit them as less dangerous wavelengths [41,42,43,44]. Nevertheless, regardless of their classification, the validation of the “blocking” ability of these sunscreens was set by ISO and FDA to have at least a critical wavelength of 370 nm when claiming the title of being a broad-spectrum sunscreen [45,46,47]. This ensures that the sunscreens on the market can cover from 280–370 nm of the sun’s wavelength. Furthermore, despite sun protective factor (SPF) being more commonly known to the public, it is, in fact, the measurement of protection against UVB. Instead, UVA is recommended to be greater than one third of the SPF value and is measured on a scale from PA+ (low) to PA++++ (high) [48,49]. As an alternative to sunscreens, the reversal or disruption of UVR-induced photoaging can also be achieved by interrupting the UVR-induced pathways in the skin. At the early stages of the UVB-induced pathway, suppressing the generation of ROS and RNS, by boosting the skin’s antioxidant defense and negating the emergence of pro-inflammatory cytokines, can aid in reducing UVR damage [50,51]. Meanwhile, hampering the production of unwanted pigmentation and encouraging the production of collagen repair at the later stages will help deter premature skin aging [52,53]. Although melanin by nature in a sense is our protector against UVR damage, through its chemical and optical filtrating properties, uneven hyperpigmentation of the skin, such as freckles, solar lentigines or melasma, are still undesirable [54,55].

Returning to the context, it has been noted that the extract from the seed has a marked aptitude as an anti-aging and photoprotective cosmetic agent (Table 2). As a photoprotective agent, ethanolic extract of the seed was reported to have a critical wavelength of 347.6 nm, when tested across the UVA-UVB spectrum. Subsequently, its hexane, ethyl acetate, and water fraction had critical wavelengths of 345, 341.6 and 362.4 nm, respectively [56]. Whilst it is not definite, the discovery proposes that the extract and fractions of the seed may contain photoprotective properties similar to sunscreen. Following this, in vitro treatment of epidermal skin (HaCaT) cells with *S. macrophylla* ethanolic extract had successfully reversed the expression of tumor necrosis factor alpha (TNF-α) by UVB, leading to the subsequent decrease in matrix metalloprotease (MMP)-1 (collagen degrading metalloprotease). Next, solvent fractionation of the ethanolic extract into both hexane and ethyl acetate fractions further enhanced its “anti-UVB” effect. The ethyl acetate fraction additionally repressed the Bcl-2 associated X protein (Bax) and nuclear factor kappa B (NF-κB), alongside TNF-α and MMP-1. This suggests that the phytocompounds that were actively attenuating the UVR-induced inflammation and cell apoptosis in the ethanolic extract can be found in the ethyl acetate fraction and, according to the solvent, is semi-polar by nature. On another note, LCMS data of the cells treated with the hexane fraction revealed a completely different effect compared to the ethanolic extract. Irradiated cells treated with the hexane fraction activated various pathways, involving the redox system, transcription to translation processes, cell growth, proliferation and migration, glycolysis, and DNA maintenance and repair, effectively attenuating the impact UVB has on human skin [57]. This ability of the *S. macrophylla* seed in reversing UVB-induced damage could be due to its antioxidant and anti-inflammatory properties. In support of these findings, several works have demonstrated that the extract of *S. macrophylla*, be it via methanol, aqueous or ethyl acetate extraction, had significant free radical scavenging and antioxidant activity [56,58,59,60]. With regard to anti-inflammation, pure phytocompounds that can be found in the seed, such as swietenine, were able to inhibit the production of nitric oxide (NO), downregulate several pro-inflammatory cytokines, and upregulate antioxidant proteins, like nuclear factor erythroid 2-related factor 2 (NRF2), and haem-oxygenase (HO-1), in lipopolysaccharide (LPS) stimulated RAW264.7 macrophage cells. The treatment of murine hepatoma (Hepa-1c1c7) cells with swietenine also induces NAD(P)H quinone oxidoreductase 1 (NQO1) dose-dependently, supporting the activation of NRF2 observed in RAW264.7 cells [61]. Other limonoids, such as swietemacrophin, 3-*O*-tigloylswietenolide, and swietemahonin E, are also potent inhibitors of inflammation. Tested against NO-generating macrophages, these phytocompounds produced an IC_50_ of less than 36.32 μM, which is suggestive of their potency in suppressing the onset of inflammation [62]. These compounds, together with humilinolide F and 3,6-*O*,*O*-diacetylswietenolide, also effectively inhibit the generation of superoxide anions in neutrophils with significant IC_50_ values of less than 45.44 μM, demonstrating their anti-inflammatory ability [62]. The same was also reported for 3-*O*-tigloyl-6-*O*-acetylswietenolide, another limonoid that is present in the seed, whereby the phytocompound effectively inhibited the generation of superoxide anion in neutrophils at an IC_50_ of 27.9 ± 2.4 μM [63,64]. Furthermore, proceranolide also showed considerable inhibition against the generation of NO in RAW264.7 macrophage cells, with an IC_50_ of 26.9 ± 0.6μg/mL, comparable to the positive control NG-methyl-L-arginine acetate salt (IC50: 13.2 ± 0.6 μg/mL) [25]. Based on these findings, this confirms the notion that *S. macrophylla* seeds contain both antioxidant and anti-inflammatory phytocompounds. Together, both these properties then function to remedy the damaging effects of UVR. Subsequently, a study further exploited the anti-inflammatory abilities of the seed [65,66]. By developing nanoemulsions and a nanoemulgel of *S. macrophylla* oil, Eid and his team achieved an even greater anti-inflammatory effect on the carrageenan-induced paw edema of rats, as compared to just the oil itself in raw form [65,66]. This sort of enhancement and modification that improves extract or phytocompound delivery to the targeted area and magnification of its bioproperties is beneficial in the cosmetic industry. It would certainly bode well, in future studies, if the research on these seed extracts could move beyond the discoveries of its bioproperties towards formulating efficient drug transport and active use of its potential in cosmetic formulation for the public.

Apart from this, other anti-premature aging properties of the seed also lie in its ability to impede the formation of irregular skin pigmentation and induce skin regeneration. In the case of skin pigmentation, one way to hinder the buildup of unwanted melanin is to inhibit the enzymatic activity of tyrosinase. Tyrosinase is a rate-limiting enzyme that converts L-tyrosine to L-3,4-dihydroxyphenylalanine (L-DOPA), which then continues through a series of conversions involving tyrosinase-related protein 1 and 2 to form melanin in the melanogenesis process [67]. Thus, the inhibition of both ethyl acetate and methanolic extract of the seed against mushroom tyrosinase suggests it might have the ability to suppress the formation of melanin [59]. Even though currently this is the only published research on the whitening effect of the seed, it is a good indicator and prompts further research regarding its anti-pigmentation properties on human tyrosinase. On another note, the wound healing properties of the seed have been explored by the authors of both [56,68]. The treatment with its ethanolic extract significantly promoted wound closure over 24 h, as tested in scratched immortalized human epithelial (HaCaT) cells. Moreover, fractionation of the ethanolic extract with water and hexane further increased its capabilities in wound closure, while the ethyl acetate fraction showed a slightly lower healing capacity, as compared to the ethanolic extract. This suggests that the extract has skin regenerative properties, and it is likely that the phytocompounds responsible are either polar or non-polar compounds [56]. Subsequently, in a study conducted by Nilugal et al. [68], an in vivo approach was used instead. Albino rats were excised on the dorsal thoracic region and ointment with ethanolic extract of the seed was applied to the wound. Wound area reduction was then measured for 20 days. The results demonstrate that the extract induced complete wound closure by day 15, instead of day 20 as observed in the negative control. In the histological images, it can be observed that the extract induced higher proliferation in the fibroblast, formed new blood capillaries, and increased collagen fibers. In short, as photoaging brings about the degradation of collagen fibers, the discovery of wound healing and skin re-epithelization properties in the seeds are promising for future cosmetic development. Tying in with the ability of the seed in inhibiting UVB-mediated production of MMP-1 (as mentioned earlier), these positive prospects should incite an increased fervor in the search for the compounds responsible for these properties. Finally, studies involving human skin and 3D culture will also increase our knowledge of the deeper mechanisms involved in the process of skin repair.

## 4. Conclusions

In summary, the potential of *S. macrophylla* seeds as a cosmeceutical product is positive. Its natural antioxidant, anti-inflammatory, antibacterial, skin whitening, and wound healing phytocompounds make it an ideal active ingredient to be present in a cosmetic product. Nonetheless, there is still a large research gap that entails the transformation of the seed extract into usable cosmetic products. Besides this, studies on skin whitening and wound healing properties of S. macrophylla are still in their rudimentary form. Furthermore, analyses on purer fractions and the phytocompounds have also not been carried out yet. On that account, additional in vivo or clinical study research to validate its effects would certainly boost its potential and development as a cosmeceutical agent.

## Figures and Tables

**Table 1:** The antimicrobial activity of *S. macrophylla* seed against various antimicrobial strains.

Seed Extract	Extraction Method	Antimicrobial Strains	Antimicrobial Activity	References
Alkaloid crude extract Rate of alkaloid extracted from the seeds: 2.85%	(A) The seeds were cleaned, sun-dried and grinded to fine powder. (B) Ethanol (300 mL; 70%) was mixed with 20 g powder of the seeds in an orbital shaker at 150 rpm for 24 h at 25 °C. (C) The extract was then evaporated with a rotary evaporator until one fifth of the initial volume was reached, before adding 20 mL of 0.1 N hydrochloric acid. (D) The extract was filtered and extracted twice with 20 mL of chloroform. (E) The extract was treated twice with 10 mL of 0.1 N hydrochloric acid and 0.1 N ammonia was added to obtain pH 9, before adding 30 mL of chloroform. (F) The operation was repeated three times, before evaporating and dissolving the residue in 20 mL of 0.02 N hydrochloric acid. (G) The extract was titrated with 0.02 N potassium hydroxide, with methyl red as an indicator.	*S. aureus* ATCC1026, *E. coli* ATCC10536, *P. aeruginosa* ATCC15442.	(A) Inhibition activity (disk diffusion method): (i) *S. aureus*: 17 mm (50 mg/mL); 21 mm (100 mg/mL); (ii) *E. coli*: 10 mm (50 mg/mL); 12 mm (100 mg/mL); (iii) *P. aeruginosa*: 12 mm (50 mg/mL); 15 mm (100 mg/mL). (B) MIC and MBC value: (i) *S. aureus*: not available (MIC); 12.5 mg/mL (MBC); (ii) *E. coli*: 25 mg/mL (MIC); 50 mg/mL (MBC); (iii) *P. aeruginosa*: 25 mg/mL (MIC); 50 mg/mL (MBC).	[16]
Methanol, ethyl acetate and butanol crude extract	The sample was air-dried, powdered and extracted with methanol, ethyl acetate and butanol.	*C. elegans* infected with MRSA ATCC33591 or methicillin-sensitive *S. aureus* (MSSA) NCTC83254.	(A) Survival of 72 h *S. aureus* infected *C. elegans* after treatment (200 μg/mL): (i) Methanol: 70% survival (3-fold change vs. untreated); (ii) Ethyl acetate: 65% (2.8-fold change vs. untreated); (iii) Butanol: 96.7% (4.2-fold change vs. untreated). (B) None of the extracts had bacteriostatic or bactericidal activity on *S. aureus* growth in vitro. (C) All three extracts significantly increased the survival of infected *C. elegans* but did not affect replication of *S. aureus*. There was no difference in CFU of the intestinal bacterial loads. (D) All three extracts promoted survival in 72 h MRSA infected *C. elegans* (>70% survival).	[21]
Methanol extract and ethyl acetate extract	(A) The sample was air-dried, powdered and extracted with methanol. (B) The crude extract was “defattened” using hexane and then further extracted with ethyl acetate.	*C. elegans* infected with *P. aeruginosa* (Bacteria strain was not specified).	(A) Ethyl acetate extract (200 and 400 μg/mL) and methanol extract (200 μg/mL) significantly improved the survival of *C. elegans* 48 h after *P. aeruginosa* infection. (B) Treatment with 200 μg/mL of ethyl acetate extract had the highest survival of 59.5 ± 1.65%. (C) MIC assay demonstrated that the ethyl acetate extract (25–1000 μg/mL) had no inhibition against *P. aeruginosa*. (D) There was no significant difference in CFU of *P. aeruginosa* in *C. elegans* intestines after both extract treatment. © Ethyl acetate extract (200 μg/mL) increased expression of lys-7 gene that was suppressed by *P. aeruginosa* infection.	[17]
Seed oil extract by diethyl ether (39%) and n-hexane (42.7%)	(A) The seeds were dried and ground to small pieces. (B) The seeds (10 g) were extracted using n-hexane and diethyl ether to recover at least 10 mL of oil using the Soxhlet apparatus. (C) The solvent was evaporated using the rotary evaporator, before further drying under the open air.	*S. aureus*, *E. coli*, *P. aeruginosa*, *Salmonella* Typhimurium. (All bacteria are in-house bacteria strains).	(A) Antibacterial activity of seed oil (disk diffusion method): (i) 10 μg/mL: 5 mm (*S. aureus*); 4 mm (*Salmonella* Typhimurium); 5 mm (*P. aeruginosa*); (ii) 20 μg/mL: 8 mm (*S. aureus*); 6 mm (*Salmonella* Typhimurium); 5 mm (*P. aeruginosa*); (iii) 50 μg/mL: 8 mm (*S. aureus*); 9 mm (*Salmonella* Typhimurium); 6 mm (*P. aeruginosa*); (iv) 100 μg/mL: 9 mm (*S. aureus*); 10 mm (*Salmonella* Typhimurium); 6 mm (*P. aeruginosa*); (v) 1000 μg/mL: 11 mm (*S. aureus*); 20 mm (*Salmonella* Typhimurium); 11 mm (*P. aeruginosa*); (vi) There was no effect on *E. coli* after the seed oil treatment.	[18]
Methanol extract	(A) The seeds were washed and dried at 50 °C for 3 days, before grinding to a coarse powder. (B) The powder was then soaked in methanol at a ratio of 1:5 (*w*/*v*) for 24 h. (C) The extract was filtered and concentrated using the rotary evaporator at 60 °C.	Gram-positive: *S. aureus*, *B. cereus*, *B. subtilis*, MRSA Gram-negative: *Proteus mirabilis*, *Yersinia* sp., *E. coli*, *Klebsiella pneumoniae*, *S. boydii*, *A. anitratus* Fungal: *Aspergillus niger*, *Microsporum fulvum*, *Rhizopus* sp. Yeast: *Candida utilis.* (Microbial strains are clinical isolates).	(A) Antimicrobial activity of methanol extract (disk diffusion method): (i) *S. aureus*: 13.0 ± 0.6 mm; (ii) *B. cereus*: 15.0 ± 0.6 mm; (iii) *B. subtilis*: 15.0 ± 1.5 mm; (iv) *S. boydii*: 15.0 ± 1.0 mm; (v) *A. anitratus*: 16.0 ± 1.2 mm; (vi) *C. utilis*: 10.0 ± 1.2 mm; (vii) No inhibitory activity was observed for MRSA, *P. mirabilis*, *Yersinia* sp., *E. coli*, *K. pneumoniae*, *A. niger*, *M. fulvum*, or *Rhizopus* sp. (B) MIC and MBC of methanol extract: (i) *B. cereus*: 3.13 mg/mL (MIC); 6.25 mg/mL (MBC); (ii) *S. aureus*: 6.25 mg/mL (MIC); 25 mg/mL(MBC); (iii) *B. subtilis*: 1.56 mg/mL (MIC); 3.13 mg/mL (MBC); (iv) *S. boydii*: 12.5 mg/mL (MIC); 25 mg/mL (MBC); (v) *A. anitratus*: 12.5 mg/mL (MIC); 25 mg/mL (MBC); (vi) *C. utilis*: 12.5 mg/mL (MIC); 25 mg/mL (MBC).	[19]
Methanol extract	(A) The seeds were dried for 2 days and pulverized to powder form. (B) The powdered seeds (30 g) were then extracted by reflux using Soxhlet apparatus for 10 h with successive organic solvent (80% methanol) and concentrated through oven drying. (C) Each fraction was collected, distilled and dried in the incubator.	Gram-positive: *S. aureus* Gram-negative: *E. coli* Fungi: *Fusarium* sp, *Helminthosporium* sp, *Alternaria* sp. (Bacteria and fungi are lab strains).	(A) Agar diffusion method (inhibition zone): (i) *S. aureus:* 20 μg/mL: 14.4 ± 1.0 mm; 30 μg/mL: 19.3 ± 0.5 mm; 50 μg/mL: 27.0 ± 1.0 mm. (ii) *E. coli:* 20 μg/mL: 21.8 ± 1.6 mm; 30 μg/mL: 31.0 ± 1.0 mm; 50 μg/mL: 40.6 ± 1.5 mm. (B) Anti-fungal activity (inhibition zone): (i) *Fusarium* sp.: 20 μg/mL: 43.3 ± 2.0%; 30 μg/mL: 54.0 ± 4.0%; 50 μg/mL: 43.3 ± 2.0%. (ii) *Alternaria* sp.: 20 μg/mL: 42.6 ± 2.5%; 30 μg/mL: 52.6 ± 1.5%; 50 μg/mL: 76.0 ± 2.0%. (iii) *Helminthosporium* sp.: 20 μg/mL: 29.3 ± 2.5%; 30 μg/mL: 37.0 ± 2.0%; 50 μg/mL: 63.3 ± 2.8%.	[20]

**Table 2:** The bioproperties and bioactivities of *S. macrophylla* seed that has cosmeceutical potential.

Seed Extract	Bioproperties	Extraction Method	In Vitro/In Vivo Studies	Bioactivity	References
Aqueous extract	Antioxidant	(A) The seeds were washed, dried at room temperature, powdered, and sieved through 40 meshes. (B) For 200 mg of powder, 1 mL of distilled water was added, before centrifuging for 15 min at 3000 rpm. (C) The supernatant was filtered and collected as the extract.	(A) Winstar rats (200–250 g) injected intraperitoneally with 65 mg/kg of streptozotocin in a volume of 1 mL/kg body weight to induce diabetes. The rats were fed with glucose 30 min prior to treatment with the extract. Blood was drawn from the tail. (B) Antioxidant testing on the extract itself.	(A) Antioxidant level in the blood demonstrated dose-dependent increase in antioxidant activity, using modified free oxygen radical defense (FORD) assay. (B) Antioxidant activity of pure aqueous extract also showed increasing antioxidant activity dose-dependently via modified FORD assay.	[58]
Aqueous extract	Antioxidant	(A) The seeds were washed, dried at room temperature, powdered, and sieved. (B) The powdered seeds were added to 200 mL of boiled distilled water, before centrifuging at 3500 rpm for 8 min and filtered.	(A) FORD assay. (B) Free radical 2,2-diphenyl-1-picrylhydrazyl (DPPH) + H2 assay. (C) Ferric-reducing antioxidant power (FRAP) assay. (D) Oxidative stress test on *Saccharomyces cerevisiae* (*S. cerevisiae*).	(A) FORD assay: increase in antioxidant activity over time. (B) DPPH assay: 56.2 ± 0.97%. (C) FRAP assay: 34.8 ± 0.13 μmol Fe + 2/g PM. (D) Oxidative stress test: I extract promotes the growth of *S. cerevisiae* over time, even in the presence of hydrogen peroxide that causes oxidative stress.	[60]
Ethyl acetate extract; methanol extract	Antioxidant and anti-pigmentation	(A) The seeds were dried and then ground to powder, before being subjected to extraction. (B) The powdered seeds were extracted with ethyl acetate and methanol in increasing order of their polarity. (C) Each extract was concentrated using a rotary evaporator at 40–50 °C.	(A) DPPH assay. (B) Tyrosinase inhibitory activity.	(A) DPPH radical scavenging capacity (500 μg/mL): (i) Ethyl acetate extract: 30.30 ± 1.63%; IC_50_ detected; (ii) Methanol extract: 56.82 ± 2.67%; IC_50_: 200 μg/mL. (B) Percentage of inhibition on tyrosinase activity: (i) Ethyl acetate extract: 14.44 ± 2.45%; (ii) Methanol extract: 15.95 ± 1.27%.	[59]
Ethanol extract (SMCE); hexane fraction (SMHF); ethyl acetate fraction (SMEAF); aqueous fraction (SMWF)	Photoprotection, antioxidant, wound healing	(A) The seeds (3 kg) were finely grounded and soaked in ethanol for 72 h at room temperature. (B) The extract was filtered and concentrated with a rotary evaporator at 40 °C to obtain SMCE. (C) SMCE was then dissolved in hexane to obtain the hexane fraction. The supernatant was dried with anhydrous sodium sulphate, before concentrating with a rotary vacuum evaporator to obtain SMHF. (D) The insoluble residues of hexane were subjected to ethyl acetate and water portioning in a 1:1 ratio. (E) The ethyl acetate fraction was dried via rotary evaporation to obtain SMEAF, while the water fraction was freeze-dried to obtain SMWF.	(A) DPPH radical scavenging assay. (B) 2′azino-bis (3, -ethylbenzothaizoline-6-sulfonic acid). (ABTS) radical scavenging assay. (C) Ferrous ion chelating assay. (D) Critical wavelength measurement. (E) Scratch wound assay on HaCaT cells.	(A) Antioxidant assays: (i) SMCE: not significant for DPPH, ABTS and ion chelating activity; (ii) SMHF: significant only for iron chelating activity at 2000 μg/mL with 14.073 ± 0.18% activity. Not significant for DPPH and ABTS assays; (iii) SMEAF: not significant for DPPH, ABTS and ion chelating activity; (iv) SMWF: (a) Significant for DPPH activity: 6.332 ± 0.80% at 2000 μg/mL; (b) Significant and dose-dependent increase for ABTS activity: 12.796 ± 2.01% at 125 μg/mL; (c) Significant and dose-dependent increase for iron chelating activity: 8.014 ± 2.51% at 125 μg/mL. (B) Critical wavelength of SMCE, SMHF, SMEAF, and SMWF are 347.6, 345, 341.6, and 362.4 nm, respectively. (C) Percentage of wound closure after 24 h of treatment: (i) SMCE (6.25 μg/mL): 54.10 ± 2.59%; (ii) SMHF (100 μg/mL): 59.45 ± 5.72%; (iii) SMEAF (12.5 μg/mL): 41.48 ± 3.91%; (iv) SMWF (50 μg/mL): 74.68 ± 5.16%.	[56]
SMCE, SMHF, SMEAF, SMWF	Photoprotection against UVB irradiation	(A) The seeds (3 kg) were finely ground and soaked in ethanol for 72 h at room temperature. (B) The extract was filtered and concentrated with a rotary evaporator at 40 °C to obtain SMCE. (C) SMCE was then dissolved in hexane to obtain the hexane fraction. The supernatant was dried with anhydrous sodium sulphate, before concentrating with a rotary vacuum evaporator to obtain SMHF. (D) The insoluble residues of hexane were subjected to ethyl acetate and water portioning in a 1:1 ratio. (E) The ethyl acetate fraction was dried via rotary evaporation to obtain SMEAF, while the water fraction was freeze-dried to obtain SMWF.	HaCaT cells treated with SMCE (6.25 μg/mL), SMHF (100 μg/mL), SMEAF (12.5 μg/mL), and SMWF (50 μg/mL) in PBS, while being exposed to 50 mJ/cm^2^ UVB. Cells were then rinsed and incubated for 24 h at 37 °C, 5% CO_2_. Protein and gene expression changes were taken 24 h post exposure. Negative control: non-irradiated cells. Inducer control: irradiated but non-treated cells.	Comparison of treatment with negative and inducer controls: (A) SMCE: (i) Gene expression changes: downregulation of TNF-α and MMP-1 (vs inducer control); (ii) Protein expression changes: downregulation of ribosomes and Filamin Bβ (vs negative control). (B) SMHF: (i) Gene expression changes: downregulation of NF-κB and cyclin D1 (vs inducer control); (ii) Protein expression changes: multiple changes across the redox system, RNA to protein processing, DNA maintenance and repair, glycolysis process, and cell growth, proliferation and migration. All changes demonstrated reversal against UVB induced damage. (C) SMEAF: (i) Gene expression changes: downregulation of TNF-α, NF-κB, MMP-1 and Bax; (ii) Protein expression changes: downregulation of PRDX-3, PDI-A3, and fascin (vs negative control). (D) SMWF: (i) Gene expression changes: no significant changes in TNF-α, NF-κB, COX-2, MMP-1, cyclin D1 and Bax; (ii) Protein expression changes: multiple changes across the redox system, RNA to protein processing, DNA maintenance and repair, glycolysis process, and cell growth, proliferation and migration, in which majority are opposite to SMHF.	[57]
Pure compounds (i) swietemacrophin (ii) humilinolide F (iii) 3,6-*O*,*O*-diacetylswietenolide (iv) 3-*O*-tigloylswietenolide (v) swietemahonin E; (vi) swietenine	Anti-inflammation	(A) Dried seeds (380 g) were pulverized and extracted with methanol for 3 days at room temperature. (B) The extract was concentrated at 35 °C with reduced pressure, before being partitioned between ethyl acetate and water in a 1:1 ratio. (C) The water fraction was further extracted with n-butanol to produce a butanol soluble fraction and water fraction. (D) The ethyl acetate fraction was further fractionated and purified to produce six pure compounds.	(A) Human neutrophils obtained from the venous blood of healthy, adult volunteers aged 20–30 years old. (B) RAW264.7 (murine macrophage) cells.	(A) Suppression of superoxide anion generation by human neutrophils (IC_50_): (i) Swietemacrophin: 45.44 ± 3.76 μM (*p* < 0.05); (ii) Humilinolide F: 27.13 ± 1.82 μM (*p* < 0.01); (iii) 3,6-O,O-diacetylswietenolide: 29.36 ± 1.75 μM (*p* < 0.05); (iv) 3-O-tigloylswietenolide: 35.58 ± 2.12 μM; (v) Swietemahonin E: 33.64 ± 2.05 μM (*p* < 0.05); (vi) Swietenine: >100. (B) Inhibition of NO generation by RAW264.7 cells: (i) Swietemacrophin: 33.45 ± 1.88 μM (*p* < 0.01); (ii) Humilinolide F: 49.36 ± 4.01 μM; (iii) 3,6-O,O-diacetylswietenolide: 64.21 ± 5.67 μM; (iv) 3-O-tigloylswietenolide: 32.62 ± 3.27μM (*p* < 0.01); (v) Swietemahonin E: 29.70 ± 2.11μM (*p* < 0.05); (vi) Swietenine: 36.32 ± 2.84.	[62]
Swietenine	Antioxidant and anti-inflammation	(A) The seeds were dried for 24 h in a drying oven at 30 °C. (B) Oil from the seeds were removed using an oil press machine. (C) The pressed seed was then sequentially extracted with hexane, ethyl acetate and methanol via a Soxhlet extractor. (D) The ethyl acetate extract was concentrated with a rotary evaporator and dried in a vacuum dryer, before further purification to isolate swietenine.	(A) RAW264.7 cells induced by lipopolysaccharide. (B) Hepa1c1c7 (murine hepatoma) cells.	(A) Swietenine dose-dependently inhibited NO production in induced RAW264.7 cells with 65.97 ± 0.7% at 0.78 μM and 21.03 ± 1.4% at 25 μM. (B) Swietenine dose-dependently significantly inhibited production of pro-inflammatory cytokine IL-1β, IFN-γ, TNF-α, and IL-6 in induced RAW264.7 cells. At 25 μM, RAW264.7 experienced a reduction in fold change in IL-1β by 1.3 ± 0.13, IFN-γ by 3.40 ± 0.07, TNF-α by 1.45 ± 0.06, and IL-6 by 1.60 ± 0.20. (C) Swietenine dose-dependently inhibited the expression of COX-2 and NF-κB of induced RAW264.7 cells. At 25μM, RAW264.7 experienced a reduction in fold change in COX-2 by 1.73 ± 0.06 and NF-κB by 2.90 ± 0.09. (D) Swietenine dose-dependently upregulated NRF2 and HO-1 in induced RAW264.7 cells. At 25μM, RAW264.7 experienced an increase in fold change in NRF2 by 2.57 ± 0.02 μM and HO-1 by 2.46 ± 0.03. (E) Swietenine induced NQO1 activity in Hepa1c1c7 cells. The CD value of swietenine was 15.8 ± 0.23 μM.	[61]
(A) *Swietenia* oil extracted from *Swietenia macrophylla* (B) Nanoemulsion *Swietenia* oil (C) Nanoemulgel of *Swietenia* oil	Anti-inflammation	Not available	Male Sprague–Dawley rats (180–200 g) with induced edema in the right hind paw. The rats were treated with the *Swietenia* oil, before being induced to have an edema.	(A) Significant dose-dependent inhibition of inflammation in the paw of the rats across 4 h of *Swietenia* oil treatment. (B) Nanoemulsion of *Swietenia* oil improved the percentage of inflammation inhibition from 54% to 76.4%, at 4 h of 4 mg/kg treatment. (C) Nanoemulgel of *Swietenia* oil improved the percentage of inflammation inhibition from 27% to 69.6%, at 4 h of 20% concentration treatment.	[65]
Ethanol extract in the form of ointment (10% *w*/*w*)	Wound healing	(A) The seeds were dried and homogenized before extracting with 95% ethanol at room temperature for 6 days. (B) The extract was filtered and concentrated.	Adult male Sprague–Dawley albino rats (200–250 g) were excised on the shaved dorsal thoracic region. The wound size was 200 mm and 2 mm deep. The wound was then blotted with a cotton swab soaked in normal saline to achieve hemostasis, before leaving it open.	(A) The wound area was closed by the ethanolic ointment by day 15 as compared to the control, which took 21 days. (B) The ethanolic ointment demonstrated higher fibroblast proliferation and increased formation of blood capillaries. There was also presence of collagen fibers and collagen deposition. As compared to the control, the control sample had disorganized fibroblasts, fewer blood capillaries and reduced collagen deposition.	[68]

## Data Availability

Not applicable.
